# Asymptomatic SARS-CoV-2 Infection Is Associated With Higher Levels of Serum IL-17C, Matrix Metalloproteinase 10 and Fibroblast Growth Factors Than Mild Symptomatic COVID-19

**DOI:** 10.3389/fimmu.2022.821730

**Published:** 2022-04-05

**Authors:** Alessandra Soares-Schanoski, Natalie Sauerwald, Carl W. Goforth, Sivakumar Periasamy, Dawn L. Weir, Stephen Lizewski, Rhonda Lizewski, Yongchao Ge, Natalia A. Kuzmina, Venugopalan D. Nair, Sindhu Vangeti, Nada Marjanovic, Antonio Cappuccio, Wan Sze Cheng, Sagie Mofsowitz, Clare M. Miller, Xuechen B. Yu, Mary-Catherine George, Elena Zaslavsky, Alexander Bukreyev, Olga G. Troyanskaya, Stuart C. Sealfon, Andrew G. Letizia, Irene Ramos

**Affiliations:** ^1^ Department of Neurology, Icahn School of Medicine at Mount Sinai, New York, NY, United States; ^2^ Center for Computational Biology, Flatiron Institute, New York, NY, United States; ^3^ Naval Medical Research Center, Silver Spring, MD, United States; ^4^ Department of Pathology, University of Texas Medical Branch, Galveston, TX, United States; ^5^ Galveston National Laboratory, Galveston, TX, United States; ^6^ Naval Medical Research Unit 6, Lima, Peru; ^7^ Department of Microbiology & Immunology, University of Texas Medical Branch, Galveston, TX, United States; ^8^ Lewis-Sigler Institute for Integrative Genomics, Princeton University, Princeton, NJ, United States; ^9^ Department of Computer Science, Princeton University, Princeton, NJ, United States; ^10^ Precision Immunology Institute, Icahn School of Medicine at Mount Sinai, New York, NY, United States

**Keywords:** SARS-CoV-2, COVID-19, asymptomatic, serum, proteomics, inflammation, innate immunity, antibodies

## Abstract

Young adults infected with SARS-CoV-2 are frequently asymptomatic or develop only mild disease. Because capturing representative mild and asymptomatic cases require active surveillance, they are less characterized than moderate or severe cases of COVID-19. However, a better understanding of SARS-CoV-2 asymptomatic infections might shed light into the immune mechanisms associated with the control of symptoms and protection. To this aim, we have determined the temporal dynamics of the humoral immune response, as well as the serum inflammatory profile, of mild and asymptomatic SARS-CoV-2 infections in a cohort of 172 initially seronegative prospectively studied United States Marine recruits, 149 of whom were subsequently found to be SARS-CoV-2 infected. The participants had blood samples taken, symptoms surveyed and PCR tests for SARS-CoV-2 performed periodically for up to 105 days. We found similar dynamics in the profiles of viral load and in the generation of specific antibody responses in asymptomatic and mild symptomatic participants. A proteomic analysis using an inflammatory panel including 92 analytes revealed a pattern of three temporal waves of inflammatory and immunoregulatory mediators, and a return to baseline for most of the inflammatory markers by 35 days post-infection. We found that 23 analytes were significantly higher in those participants that reported symptoms at the time of the first positive SARS-CoV-2 PCR compared with asymptomatic participants, including mostly chemokines and cytokines associated with inflammatory response or immune activation (i.e., TNF-α, TNF-β, CXCL10, IL-8). Notably, we detected 7 analytes (IL-17C, MMP-10, FGF-19, FGF-21, FGF-23, CXCL5 and CCL23) that were higher in asymptomatic participants than in participants with symptoms; these are known to be involved in tissue repair and may be related to the control of symptoms. Overall, we found a serum proteomic signature that differentiates asymptomatic and mild symptomatic infections in young adults, including potential targets for developing new therapies and prognostic tests.

## Introduction

In March 2020 the World Health Organization declared the Coronavirus Disease 2019 (COVID-19) a global pandemic (1). As of March 2022 there have been roughly 445 million cases and more than 5.9 million deaths reported worldwide (2). SARS-CoV-2 is highly transmissible (3, 4) and the mortality rate is reported to be between 0.9 to 7.7%, depending on the country (5). Severe acute respiratory syndrome coronavirus 2 (SARS-CoV-2) frequently causes mild or asymptomatic disease, especially in young individuals (6–8) who contribute to viral transmission.

Most studies on the pathogenesis of COVID-19 have focused on severe cases [e.g (9–12)]. Although the immune response of individuals with asymptomatic and mild disease has been studied (13–16), it is much less well characterized. Indeed, many important studies characterized the immune response to severe COVID-19 by comparing to mild cases and uninfected participants (12, 17, 18). Studies of mild COVID-19 and asymptomatic SARS-CoV-2 infections have the potential to identify correlates of protection from severe disease, which could indicate new targets for therapy and prognostic tests.

Individuals with asymptomatic infections develop SARS-CoV-2 antibodies, but the magnitude of their response is lower than those with moderate to severe disease (19, 20), and similar to those with mild disease (6). On the other hand, we also learned from elegant studies that asymptomatic individuals are able to mount an efficient memory T cell response against SARS-CoV-2 during the convalescent phase (14, 15). However, the extent and characteristics of the immune response during the acute phase of the disease in asymptomatic individuals remains unclear.

Individuals with mild COVID-19 produce several of the pro-inflammatory mediators seen in individuals with severe disease, including IL-6, CXCL10, TNF-α, MCP-1 and IFN-*γ* (12, 21), but a prolonged duration of the inflammatory response is likely characteristic of severe cases (9). In this sense, a longitudinal immune response profile of asymptomatic and symptomatic individuals is needed to better understand their differences and the mechanisms that protect some individuals from developing symptoms.

Here, we characterized the dynamics of the early humoral and innate immune response in otherwise healthy young adults with asymptomatic or mildly symptomatic SARS-CoV-2 infection, in a subset of participants of the previously reported COVID-19 Health Action Response for Marines (CHARM) cohort study (22). The study design of the CHARM cohort, with regular antibody and PCR testing, allowed for collection of pre-infection samples, approximate identification of the beginning of the infection, and follow-up sampling of the infected participants for up to 63 days after infection in this subset.

We found similar levels of induction of SARS-CoV-2 spike (S)-specific IgG and IgM antibodies in asymptomatic and symptomatic participants, as well as similar neutralizing antibody levels. Slightly higher viral load, as approximated by Ct value, was found in symptomatic participants as compared to asymptomatic at first positive PCR (PCR+) detected. Longitudinal proteomic analysis of 92 analytes in serum revealed a subset of pro- and anti-inflammatory markers that are positively correlated with symptoms and viral load, as well as others that are exclusively associated only with either number of symptoms or with viral load. Interestingly, we found a proteomic signature associated with asymptomatic infections, that includes the analytes IL-17C, MMP-10 and FGF-23, with previously described functions in tissue repair. Overall, our findings suggest that control of symptoms during SARS-CoV-2 asymptomatic infections, as compared to mildly symptomatic infections, could be achieved by the appropriate balance of inflammatory and tissue repair associated mediators in young adults

## Methods

### Cohort and Data Collection

The CHARM cohort study, which has been previously described (22), was designed to identify SARS-CoV-2 infection regardless of symptoms and to assess the host immune response during acute infection and early convalescence stages. The cohort is composed of Marine recruits that arrived at Marine Corps Recruit Depot—Parris Island (MCRDPI) for basic training between May and November 2020, after undergoing two quarantine periods. The first one was a home-quarantine, and the second a supervised quarantine starting at enrollment in the CHARM study, as previously described (22, 23).

At enrollment, participants completed a questionnaire consisting of demographic information, risk factors, reporting of 13 specific COVID-19-related symptoms or any other unspecified symptom since the previous visit, or in the previous 2 weeks in the case of the first visit or if more that 2-weeks since the last visit had passed, temperature recording and brief medical history. At approximately weeks 0, 1, 2, 4, 6 and 8 after enrollment, additional PCR testing was performed, and the follow-up symptom questionnaire was administered. Serum samples were collected in all the visits. For the analysis presented in this study, we included a subset of participants who were PCR negative and SARS-CoV-2 seronegative at enrollment (negative for IgG RBD and S titers, at a threshold titer of 1:150) (22), had zero (negative controls) or at least one PCR detected (infected participants), and had sera available after PCR detection in the case of infected participants.

### Collection of Biological Specimens and Quantitative PCR Testing

At each time point, blood was collected using serum separator tubes (SST) which were centrifuged to isolate serum (1500 x g for 10 min). Aliquots of serum were frozen at -80°C. Nares swabs were collected and kept at 4°C for SARS-CoV-2 PCR testing. All PCR assays were performed within 48 h of sample collection at the high complexity Clinical Laboratory Improvement Amendments-certified laboratories, Lab24Inc (Boca Raton, FL, USA, assays performed May 11-Aug 24, 2020) and the Naval Medical Research Center (Silver Spring, MD, USA, assays performed Aug 24 -Nov 2, 2020), using the US Food and Drug Administration-authorized Thermo Fisher TaqPath COVID-19 Combo Kit (Thermo Fisher Scientific, Waltham, MA, USA).

### Enzyme-Linked Immunosorbent Assay for Evaluation of SARS-CoV-2 RBD Specific IgG and IgM Titers

IgG and IgM SARS-CoV-2 specific antibodies in serum were evaluated using an enzyme-linked immunosorbent assay (ELISA) as previously described (22, 23). 384-well Immulon 4 HBX plates (Thermo Fisher Scientific, Waltham, MA, UA), were coated overnight at 4°C with 2 μg/mL of recombinant His-tagged spike receptor-binding domain (RBD) (Sino Biological, Beijing, China) or spike (S) protein (LakePharma, Irving, TX, USA). Plates were washed with 0.1% Tween-20 using an automated ELISA plate washer (AquaMax 4000, Molecular Devices, San Jose, CA, USA), and blocked for 1 h at room temperature with 3% milk in PBS-T. Blocking solution was removed, and serum samples diluted in 1% milk PBS-T were dispensed in the wells. At least two positive controls (sera with known IgG presence), eight negative controls (sera collected before July 14, 2019), and four blanks (no serum) were included in every assay. Plates were incubated for 2 h at room temperature, and then washed. Next, peroxidase conjugated goat F(ab’)2 Anti-Human IgG (Abcam, Cambridge, UK) was added at 1:5000–1:10 000 dilutions (determined after optimization for each antibody lot) in 1% milk PBS-T, and plates were incubated for 1 h at room temperature. Plates were washed, developed using o-phenylenediamine, and the reaction was stopped after 10 min with 3M HCl. Optical density (OD) at 492 nm was measured using a microplate reader (SpectraMax M2, Molecular Devices). All serum samples were screened at a 1:50 dilution with RBD. Those samples with an OD 492 nm value higher than the average of a set of 8 negative controls plus three times their SD in the screening assay underwent titration assay (six serial 1:3 serum dilutions starting at 1:50) using S protein. Serum samples were considered positive for each assay when at least two consecutive dilutions showed higher OD 492 nm than the average of the negative controls plus three times their SD at the correspondent dilution or 0·15 OD 492 nm. Specificity was 100% on both RBD and S protein ELISA using 70 control sera obtained before July 14, 2019. At baseline, participants were only considered seropositive to SARS-CoV-2 if IgG titrations for both S and RBD ELISA gave a positive result at a minimum of 1:150 dilution.

### Neutralization Assays

Studies involving infectious SARS-CoV-2 were performed at the Galveston National Laboratory as previously described (6). Two-fold serial dilutions of heat-inactivated serum at an initial dilution of 1:20, were prepared in serum free media (Minimum Essential Medium; Thermo fisher Scientific, containing 25 mM HEPES and 0.05 g/L Gentamicin sulfate) and incubated with an equal volume of mNeonGreen SARS-CoV-2 (24) for 1 hour at 37°C (200 plaque forming units/well, which results in a final multiplicity of infection of 0.005) in humidified 5% CO_2_. Virus-serum mixtures were then added to Vero-E6 monolayers in 96 well optical black plates and incubated at 37°C. Plates were read using the BioTek Cytation 5 plate reader (EX 485 nm, EM 528 nm) at 48 h post-infection. Following background signal correction, virus neutralization half-maximal inhibitory serum dilution (ID50) values were determined using a 4-parameter logistic regression.

### Proteomics Analysis Using OLINK Proximity Extension Assay

For proteomics, we used the commercially available Inflammatory panel from OLINK®, composed of 92 analytes. PEA was performed at the Human Immune Monitoring Center at Mount Sinai, New York, as previously described (25). Briefly, sera samples were inactivated by UV exposition for 1 h and mixed with PEA probes that are oligonucleotide-labeled antibodies used to bind to target proteins. Then, a combined extension and pre-amplification mix of reagents were added to the samples incubated, with PEA probes allowing subsequent extension by a DNA polymerase. Upon binding to the protein epitope, the paired oligonucleotide sequences are amplified through a quantitative real-time PCR (qRT-PCR) reaction. The results are shown as NPX (Normalized Protein eXpression), that is an arbitrary unit which is Log2 scale and is calculated from Ct values generated by the qRT-PCR reaction, and after data pre-processing is performed to minimize inter and intra-assay variation. The data were pre-processed by Olink using NPX Manager software. For longitudinal analysis, samples from the different time points were grouped in the following categories: “Before” infection, “First PCR+”, “3-10 days”, “11-21 days”, “22-35 days”, and “> 35 days” after infection.

### RNA-Seq Processing and Analysis

Total RNA from PAXgene preserved blood was extracted using the Agencourt RNAdvance Blood Kit (Beckman Coulter) on a BioMek FX^P^ Laboratory Automation Workstation (Beckman Coulter). Concentration and integrity (RIN) of isolated RNA were determined using Quant-iT™ RiboGreen™ RNA Assay Kit (Thermo Fisher) and an RNA Standard Sensitivity Kit (DNF-471, Agilent Technologies, Santa Clara, CA, USA) on a Fragment Analyzer Automated CE system (Agilent Technologies), respectively. Subsequently, cDNA libraries were constructed from total RNA using the Universal Plus mRNA-Seq kit (Tecan Genomics, San Carlos, CA, United States) in a Biomek i7 Automated Workstation (Beckman Coulter). Briefly, mRNA was isolated from purified 300 ng total RNA using oligo-dT beads and used to synthesize cDNA following the manufacturer’s instructions. The transcripts for ribosomal RNA (rRNA) and globin were further depleted using the AnyDeplete kit (Tecan Genomics) prior to the amplification of libraries. Library concentration was assessed fluorometrically using the Qubit dsDNA HS Kit (Thermo Fisher), and quality was assessed with the Genomic DNA 50Kb Analysis Kit (DNF-467, Agilent Technologies). Following library preparation, samples were pooled, and preliminary sequencing of cDNA libraries (average read depth of 90,000 reads) was performed using a MiSeq system (Illumina) to confirm library quality and concentration. Deep sequencing was subsequently performed using an S4 flow cell in a NovaSeq sequencing system (Illumina) (average read depth ~30 million pairs of 2×100 bp reads) at New York Genome Center.

All RNA-seq data was processed in a uniform pipeline. Gene expression levels were quantified with kallisto (v0.46.0) (26), using Gencode v34 transcript annotations (27). Transcript-level quantifications were aggregated to gene level using the tximport (v1.14.2) package, and expression levels were normalized across samples using DESeq2 (28). Differential gene expression analysis was performed with DESeq2, comparing samples during the various time points during infection to baseline gene expression levels, controlling for sex and plate number to minimize batch effects. Immune cell type proportions were estimated from bulk RNA-seq using CIBERSORTx (29). In order to obtain total proportions of each major cell type, multiple cell subsets were combined by adding the component proportions (e.g. resting and activated natural killer (NK) cell categories were summed up to a single NK cell type category).

### Statistics

Statistical analysis was performed with Rstudio (version 1.3.1093), R (version 4.0.2) and the Prism 9 software. Correlations between symptoms and Ct were evaluated using the Pearson’s method. Distribution of ethnicity, race and sex among study groups was assessed with a Pearson’s Chi-squared test followed *post-hoc* analysis based on residuals, adjusted using the Bonferroni method. Serological and Ct pairwise comparisons between Asymptomatic and Symptomatic groups were performed using the non-parametric Mann-Whitney U test.

For the PEA analysis, delta NPX (ΔNPX) values were obtained for every participant by subtracting the NPX value at baseline (before infection) from the NPX value at every time point after detection by PCR (first PCR+ and later). This implicitly controls for differences between individual baselines, allowing us to compare only the differences observed in each participant during infection, rather than using a different population as a healthy control which introduces many confounding factors. Distributions of ΔNPX values were compared using the non-parametric Mann-Whitney U test and corrected for multiple hypothesis testing by the Benjamini-Hochberg procedure at an FDR of 0.05. Of the 92 analytes measured, 66 showed any significant changes from baseline at any point during or after infection, so only these 66 analytes with differential activity were studied further. Correlations between NPX values and both number of symptoms and Ct values were computed using linear mixed models (LMMs), with the predicted slope (LMM coefficient) representing the direction and degree of correlation.

### Study Approval

The CHARM study was approved by the Naval Medical Research Center (NMRC) institutional review board (IRB), protocol number NMRC.2020.0006, in compliance with all applicable U.S. federal regulations governing the protection of human participants. Research performed at Icahn School of Medicine at Mount Sinai (ISMMS) as part of this study was reviewed by the ISMMS Program for Protection of Human Participants and the Naval Information Warfare Center Pacific (NIWC Pacific) Human Research Protection Program (HRPP) and received non-human participants (NHS) determination. All participants provided written informed consent.

## Results

### Cohort Description, Symptoms, Viral Load, and Antibody Responses

The CHARM cohort study has been previously described (22, 23). With the purpose of investigating the dynamics of the early immune response in asymptomatic and symptomatic participants, we selected a subset of participants that i) were seronegative and SARS-CoV-2 PCR negative at enrollment in CHARM and ii) were PCR negative during the entire study (negative controls, n=23), or were PCR positive at least at one time point during the study (infected participants, n=149). Within the infected participants, we defined two groups: the Asymptomatic group (n=85) included participants that, between the time of diagnosis and the end of the study, reported 0 or 1 symptoms total and temperature below 100.4°F; the Symptomatic group (n=64) included participants that, between the time of diagnosis and the end of the study, reported more than 1 symptom total and/or temperature above 100.4°F. We identified 4 participants that had one symptom at one time point within the first 2 weeks after the first PCR+ (1 with headache, 1 with chill and 2 with loss of taste), and they were included in the Asymptomatic group. Therefore, the initial sample population consists of 172 participants, selected following the above criteria and included in this study in their order of enrollment and based on availability of samples, of which 88.3% reported as being males, and the age mean was 19.57 ± 2.22 years. Race and Ethnicity distribution was balanced across the participants in the Negative Control, Asymptomatic and Symptomatic participants (**Table 1**, p-values = 0.47 and 0.21 for race and ethnicity, respectively). However, in agreement to our findings in a separate sub-study within CHARM (6), male participants were more represented than female participants in the Asymptomatic group as compared with the rest of the groups (*post-hoc* adjusted p-value = 0.03).

**Table 1:** Contingency table showing the distribution of sex, race, and ethnicity in the study groups.

	Negative Control	Asymptomatic	Symptomatic
**Sex**			
**F (11.6%)**	4 (17.4)	4 (4.7)*	12 (18.7)
**M (88.4%)**	19 (82.6)	81 (95.3)*	52 (81.3)
	Chi-squared p-value = 0.02; * post-hoc p-value = 0.03		
**Race**			
**White (73.3%)**	16 (69.6)	62 (72.9)	48 (75)
**Black (12.2%)**	2 (8.7)	11 (12.9)	8 (12.5)
**Asian (2.9%)**	0 (0.0)	1 (1.2)	4 (6.25)
**American Indian/Alaska Native (1.7%)**	0 (0.0)	1 (1.2)	2 (3.1)
**Multi-racial (2.9%)**	1 (4.3)	4 (4.7)	0 (0.0)
**Native Hawaiian/Other Pacific Islander**	0 (0.0)	0 (0.0)	0 (0.0)
**Other (0.6%)**	0 (0.0)	0 (0.0)	1 (1.56)
**Non-specified (6.4%)**	4 (17.4)	6 (7.1)	1 (1.56)
	Chi-squared p-value = 0.47		
**Ethnicity**			
**Hispanic (33.72%)**	6 (26.1)	33 (38.8)	19 (29.7)
**Non-hispanic (40.7%)**	11 (47.8)	29 (34.1)	30 (46.9)
**Non-specified (25.58%)**	6 (26.1)	23 (27.1)	15 (23.4)
	Chi-squared p-value = 0.21		

**Figure 1:**
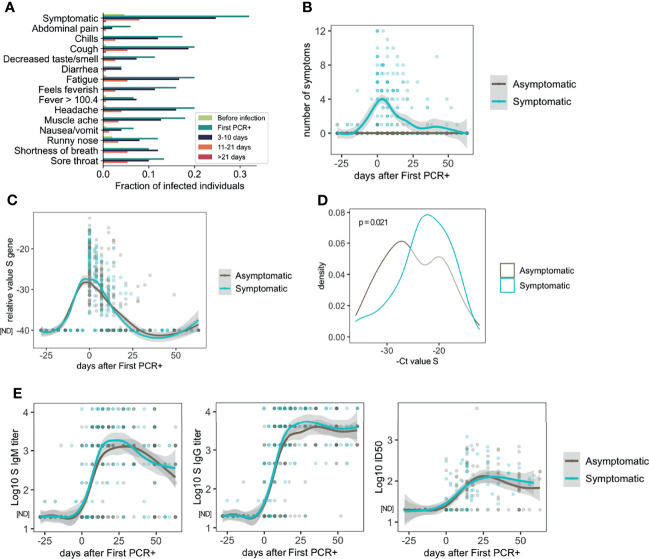
Symptoms, viral load and antibody response in asymptomatic and mild symptomatic participants (n=85 asymptomatic and n=64 symptomatic). **(A)** Distribution of symptoms and fever reported over time. **(B)** Number of symptoms over time. **(C)** Longitudinal distribution of viral load as measured by PCR (S Ct values). **(D)** S gene PCR results at first SARS-CoV-2 positive test in asymptomatic and symptomatic participants. **(E)** Longitudinal analysis of serum IgM and IgG S-specific titers (n=85 asymptomatic and n=64 symptomatic participants), and half inhibitory infectious dose (ID50), (n=45 asymptomatic and n=46 symptomatic participants). ND, Not Detected.

The distribution of symptoms over time among all participants in which infection was detected is represented in **Figure 1A**. All the 13 symptoms and temperature measurements were more frequently reported at the time of first PCR+ (**Figure 1A**), while less frequency was found at 3-10 and 11-21 days after first PCR+. Most of the symptomatic participants resolved all symptoms by 21 days after infection (**Figures 1A, B**).

Symptomatic and asymptomatic participants showed similar dynamics of viral load as measured by PCR for the S gene (**Figure 1C**), or the N or ORF1ab genes (**Figure S1A**). However, comparison of the PCR measurements at the time of the first PCR+, indicated that symptomatic participants had lower S Ct values (22.91 ± 5.05) and therefore higher viral load, than asymptomatic participants (24.96 ± 5.67, p=0.021) on average (**Figure 1D**). Results for N gene (23.38 ± 5.88 symptomatic and 24.81 ± 5.76 asymptomatic) and ORF1ab gene (23.13 ± 6.67 symptomatic and 24.77 ± 5.49 asymptomatic) Ct analysis at the time of first PCR+ followed a similar trend but did not yield statistically significant differences (**Figures S1A, B**; p = 0.095 and p = 0.0528, respectively).

Since it is known that there are sex differences in the immune response and disease manifestation due to SARS-CoV-2 infection (30), we analyzed the Ct values at the time of diagnosis in asymptomatic and symptomatic males and females. Interestingly, while symptomatic males showed significantly higher viral load than asymptomatic males for the three genes (S, N and ORF1ab), no differences were found in females regarding symptoms for any of the genes (**Figure S1C**). It is important to note that the number of females included in this analysis (4 symptomatic and 12 symptomatic) was notably lower than males, therefore the lack of significance in this comparison in females could be due to limited statistical power from small sample size data.

Then, we assessed the dynamics of the antibody response to SARS-CoV-2 infection. S-specific IgG and IgM were measured in longitudinal serum samples from symptomatic (n=55) and asymptomatic (n=84) participants. As shown in **Figure 1E**, there was high variability in the antibody titers among the participants. However, very similar longitudinal profiles were observed overall when we compared asymptomatic and symptomatic participants. Similar observations were found when we analyzed the virus neutralizing activity of the serum from a subset of participants (n=34 symptomatic and n=44 symptomatic participants, **Figure 1E**). We did not find statistically significant differences between asymptomatic and symptomatic participants when the last time point with serum available for each participant (collected 10-63 days after first positive PCR) was compared for either IgG titers (3,133; 95% CI 2415-4044 asymptomatic, and 4,295; 95% CI 3342-5534 symptomatic group) or ID50 values (92.0; 95% CI 66.6-127.4 asymptomatic and 100.0; 95% CI 71.1-140.6 symptomatic) (**Figure S1D**). No differences were found regarding sex in the levels of S-IgG specific titers or neutralizing activity (**Figure S1E**).

### Proteomic Profiling Shows Three Temporal Waves of Immune Mediators That Are Resolved Early After Infection in Asymptomatic and Mild COVID-19

**Figure 2:**
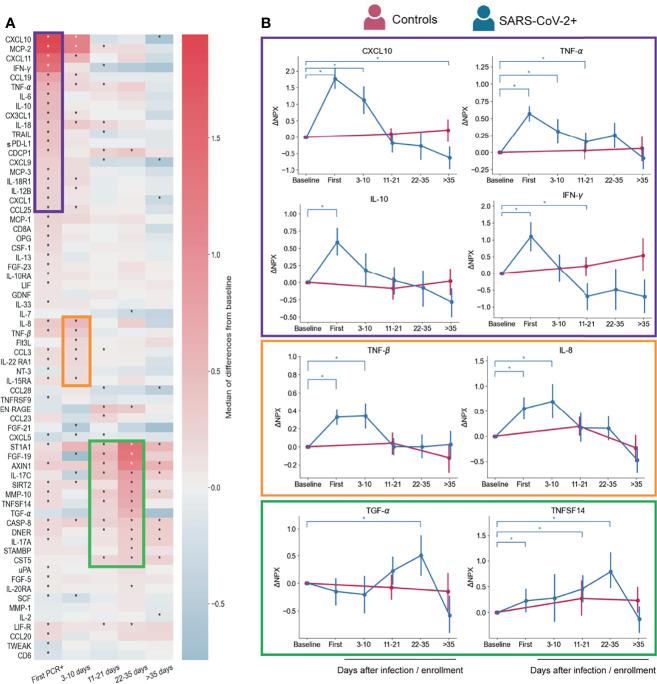
Serum proteins measured by PEA with overall changes over time with respect to pre-infection (n=88 participants) regardless symptoms status. **(A)** Heatmap showing the proteomic signature with relative expression of the markers with significant changes overtime in infected participants. **(B)** Representative temporal profile of markers belonging to the first, second and third wave (represented by the boxes in purple, orange and green, respectively). Controls in panel B are uninfected participants (n=23) that were included in the analysis with samples collected at study enrollment (baseline), 14 days, and 56 days after enrollment. Mean and 95% CI are indicated. *p < 0.05.

We performed a longitudinal Proteomic Extension Assay (PEA) on the sera of 89 infected participants (42 symptomatic and 47 asymptomatic). We also included 23 participants with no positive SARS-CoV-2 PCR test at any time point and no evidence of antibodies from previous infections as controls. First, we evaluated changes overtime of the 92 markers in the PEA panel, considering all infected participants. Normalized Protein eXpression (NPX) values for each participant were analyzed as ΔNPX (NPX sample each time point – NPX baseline) (**Table S1**). The values compared are therefore always the differences in a specific participant from their own pre-infection healthy baseline. A longitudinal proteomic analysis until 60 days post first SARS-CoV-2 PCR+ showed significant changes over time for 66 of the markers from this panel (**Figure 2A**). By sorting these markers according to their time of maximum value, we identified three temporal waves of inflammatory and immunoregulatory mediators upregulated after infection (**Figures 2A, B**).

The first wave started at the time of first PCR+, coinciding with when most symptoms were reported (**Figure 1A**). As expected, this group of immune mediators is composed of inflammatory cytokines, such as IFN-*γ*, IL-12B, TNF-α, IL-18, IL-6, and the chemokines CXCL10, MCP-2, CXCL11, and CX3CL1, consistent with previous reports (17, 21, 31, 32) (**Figures 2A, B**). We also detected immunoregulatory markers, such as IL-10, which was previously reported as a marker of COVID-19 severity (33), and soluble PD-L1 (sPD-L1), which binds to PD-1 on the surface of effector CD8 T cells promoting their suppression or exhaustion (34, 35). IL-18 upregulation may indicate inflammasome activation, however, we did not detect an increase of IL-1β, a cytokine that is also part of this pathway (36).

The second wave (**Figures 2A, B**) is composed by a slight upregulation of relatively few mediators including IL-8, CCL3, TNF-β, Flt3L, IL-22RA1 and IL15RA. Levels of these markers showed already higher expression at first PCR+ time point with respect to their baseline, but peaked at 3-10 days after infection. IL-8 and CCL3 which have been reported as COVID-19 severity markers (21, 37–39) and TNF-β was found in less severe disease (9), which is in agreement with our findings.

Though none of the participants in this study developed severe COVID-19 and many of them resolved symptoms within days after first PCR+ (**Figure 1A**), we observed upregulation of markers of severity early during infection. However, most of those markers, represented in the first and second waves, returned to baseline levels within the first 10-35 days of diagnosis (**Figure 2A**), indicating a rapid control of the systemic inflammatory responses in this cohort.

Interestingly, the third wave (11-35 days post first PCR+) is composed of some mediators that were induced already at the time of first PCR+, but peaked at later time points (22-35 days), when most of the participants have cleared the virus (**Figures 2A** and **1C**). TGF-α and FGF-19 may be indicators of tissue repair related to the infection (**Figures 2A, B**) (40–42). One of the markers of this group is ST1A1 which plays a role in acetaminophen metabolism (43) but the cause of its increase in circulation is unknown. Levels of IL-17A (**Figure 2A**) and TNFSF14 (**Figures 2A, B**) are enhanced early after infection and gradually increase until late time points. Both proteins have been described as markers of severe cases (11, 17, 44), although our cohort is composed of only mild and asymptomatic cases. Proteins from the third wave were likely induced by early mediators in the acute phase of the infection, and might contribute to the recovery from infection or disease since their peak of expression coincides with clearance of virus and symptoms (**Figures 1A, B**).

To further understand the inflammation serum dynamics, we utilized a blood RNA-seq dataset that was generated as part of the CHARM study to estimate proportions of circulating innate immune cells. Interestingly, proportions of monocytes, dendritic cells (DC) and NK cells were increased at the time of first PCR+ with respect to the baseline levels (**Figure S2**), which coincides with the first wave of inflammatory markers in our PEA analysis (**Figure 2**). DC and NK cells proportions were also significantly elevated at the 3-10 days time points. Chemo-attractants of NK cells, DC and monocytes (e.g. CXCL10, MCP-1, 2 and 3) (45, 46) were detected in serum as part of the first and second waves (**Figure 2**) and could explain the increased cell proportions detected by RNA-seq analysis (**Figure S2**).

### Serum Immune Mediators Correlate With Number of Symptoms and Viral Load

**Figure 3:**
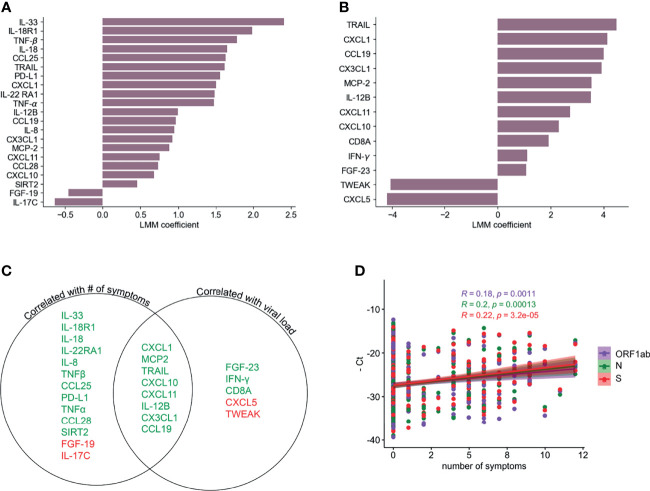
Correlation of PEA serum markers with symptoms and viral load. LLM correlation analysis of PEA detected markers and number of symptoms **(A)** and relative viral load determined as the average of the negative Ct values of S, N and ORF1ab genes **(B)** FDR cutoff = 0.05. **(C)** Venn diagram showing the serum markers that are correlated with number of symptoms and/or viral load. Green denotes positive correlation and red denotes negative correlation. **(D)** Correlation (Pearson’s) between PCR negative Ct values for ORF1ab, N and S genes and numbers of symptoms.

We next used a linear mixed model (LMM) to investigate the relationship between the inflammatory profile and the number of symptoms or viral load. **Figure 3A** shows 19 analytes that are positively and 2 that are negatively correlated with the number of symptoms at the same time point. In the case of viral load, we found 11 analytes that were positively correlated and 2 that were negatively correlated (**Figure 3B**). Interestingly, there were 8 markers, most of them chemokines, which were positively correlated with both number of symptoms and viral load (**Figure 3C**). Related to this, we found a weak but significant positive correlation (Pearson’s), between the number of symptoms and viral load (-Ct values for genes S, N or ORF1ab) (**Figure 3D**). CXCL10, CXCL1, CXCL11, CX3CL1 were correlated with severity in previous reports (17, 21) but showed a rapid decline over time in the participants of this cohort (**Figure 2A**), similarly to the monocyte chemoattractant MCP-2, IL-12B and the death-receptor ligand TRAIL, all of which are known to be important for viral clearance (47, 48). None of the analytes that correlated only with the number of symptoms showed any trend towards significance with viral load (**Figure 3C**). Among those that were significantly correlated with viral load, IFN-γ was the only one that showed some level of correlation with the number of symptoms as well, which was significant only before multiple hypothesis correction (p= 0.000637). The positive correlation between viral load and IFN-γ is in agreement with other reports showing the importance of this cytokine in promoting direct and indirect anti-viral immunity (49).

### Dynamics of Serum Immune Signatures Are Associated With Onset of Symptoms

**Figure 4:**
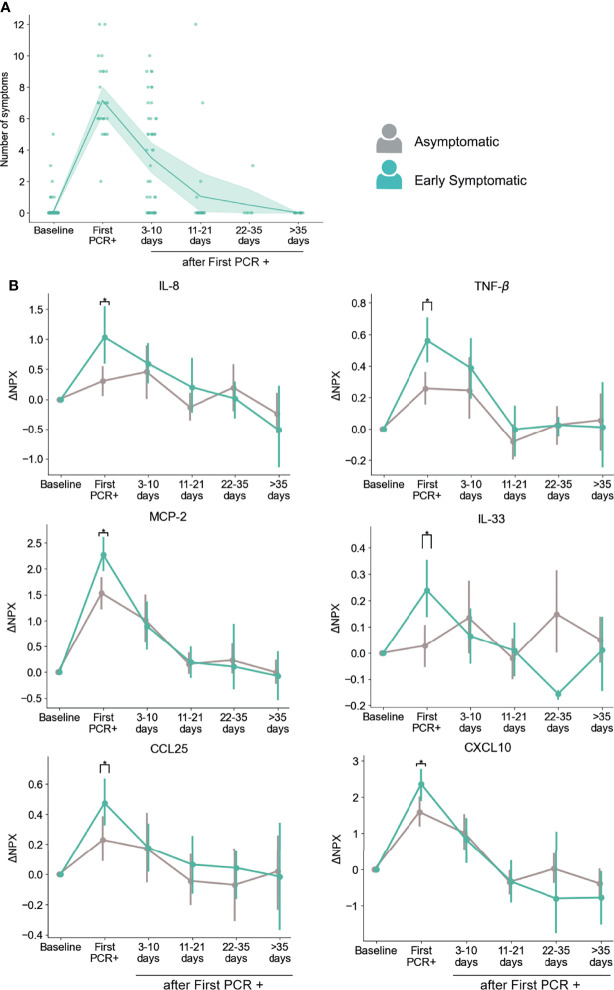
Dynamics of serum markers with higher upregulation in Early Symptomatic participants (symptoms reported at First PCR+) than in Asymptomatic participants. **(A)** Temporal distribution of the number symptoms in Early Symptomatic participants. **(B)** Representative markers that are significantly upregulated in Early Symptomatic in comparison to Asymptomatic group of participants. Mean and 95% CI are indicated. *p < 0.05.

In order to understand the longitudinal inflammatory response induced by SARS-CoV-2 infection during symptomatic and asymptomatic infections, we analyzed the differences between these groups of participants over time. Our initial analysis did not identify significant differences for any of the cytokines between those two groups after correcting for multiple hypothesis testing. However, we found a strong association between the number of symptoms at a given time point and the inflammatory landscape in serum (**Figure 3A**), which suggests that the longitudinal analysis could be obscured by the fact that symptoms do not occur at the same time (with respect to first PCR+ detection) in all participants. Therefore, we further stratified the group of Symptomatic participants according to symptom onset: the Early Symptomatic group includes those participants that reported more than 1 symptom at their time of first PCR+ (n=31, **Figure 4A**), and the Late Symptomatic group includes those that reported more than 1 symptom for the first time 3 or more days post first PCR+ (n=15) (**Figure S3A**).

Using this approach, we found that participants in the Early Symptomatic group presented 23 analytes with higher levels of upregulation than the Asymptomatic group (**Table S2**), and these differences were only detected at the first PCR+ time point, when they first reported symptoms. Among those analytes, we found the pro-inflammatory mediators CXCL10, CCL25, MCP-2, IL-8, TNF-β and the alarmin IL-33 (**Figure 4B**). We did not find significantly higher levels of induction of these analytes between the Late Symptomatic and the Asymptomatic group (**Figure S3B**). It is important to note that the Late Symptomatic group was composed of a smaller group of participants (n=15) and was very heterogeneous with regards to the time of occurrence of symptoms (**Figure S3A**), with some participants peaking at 3-10 days after first PCR+ while others peaked at 11-21 days. In addition, the Late symptomatic group tended to report on average fewer symptoms (4.89) than the Early Symptomatic group (7.15) at their peak of their symptoms (3-10 days post first PCR+ and first PCR+, respectively). This is reflected in the high variance of the presence of these analytes over time in serum (**Figure S3B**). Therefore, the Early Symptomatic group represents a clearer temporal distribution and larger sample size to compare with the Asymptomatic group. Even with the high variability found in the Late Symptomatic group, we observed several mediators, including IL-8, MCP-2, and CXCL10, that peaked 3-10 days post-first PCR+, coinciding with the time of the maximum average number of symptoms in these participants (**Figure S3B**).

To further assess the co-occurrence with symptoms for these analytes, we did an additional longitudinal analysis, in which we evaluated their levels with respect to the time of maximum number of symptoms reported by each participant. As shown in **Figure S4A**, they all peaked at the time of reporting of the maximum number of symptoms and started to decrease early after this timepoint, except for TNF-β, that was maintained longer (at 3-10 days after maximum of symptoms), and then decreased (**Figure S4A**).

In addition, we performed an independent analysis in which we included PEA data from all the PCR+ timepoints from all symptomatic participants (both Early and Late Symptomatic) at the time they experienced symptoms, and from all PCR+ timepoints in the case of the asymptomatic participants, regardless of the time after infection. CXCL10, CCL25, MCP-2, IL-8, TNF-β, TNFSF14, and IL-33 showed significantly increased levels in participants reporting symptoms at the time of sampling than in asymptomatic participants at any PCR+ time point (**Figure S4B**). Therefore, we identified multiple inflammatory mediators associated with the presence of symptoms, where their peaks coincide with the time of maximum number of symptoms reported and decrease over time as symptoms clear.

A total of 6 proteins were found to be significantly higher in females than males (TNFSF14, AXIN1, SIRT2, CASP-8, ST1A1, TRANCE) in both Asymptomatic and Symptomatic groups, in an analysis that included all samples with SARS-CoV-2 PCR+ results (**Figure S5**). Of those, AXIN1, SIRT2, ST1A1 and TRANCE showed significant upregulation in symptomatic participants only in males. However, assessment of differences with regards to presence of symptoms in females is challenging due to their low numbers in this analysis (n=16 total, n=12 with detected SARS-CoV-2 infection).

We next assessed the estimated cell proportions in the blood RNA-seq dataset in Asymptomatic and Early Symptomatic participants. Early Symptomatic participants showed significantly higher frequencies of DC at the first PCR + time point and of NK cells at 3-10 days after first PCR + (**Figure S6**) as compared to Asymptomatic participants. Interestingly, we also found significantly higher proportions of monocytes, DC, and NK cells in Early Symptomatic than in Asymptomatic participants at 22-35 days after infection. However, we did not find any significant differences at 22-35 days between these groups for any serum markers at these time points, so the connection between this increase in the proportion of these immune cells and the cytokine profile between asymptomatic and symptomatic participants is unclear. A lower proportion of neutrophils was found in the Early Symptomatic group than in the Asymptomatic group at 3-10 days after infection. A previous report found similar results when they analyzed mature neutrophils by flow cytometry, but an opposite trend in the case of immature neutrophils (16). Therefore, it is possible that the estimated neutrophil proportions in this bulk-RNA analysis correspond to mature neutrophils.

### Candidate Markers That Could Give Insights on Suppression of COVID-19 Related Symptoms

**Figure 5:**
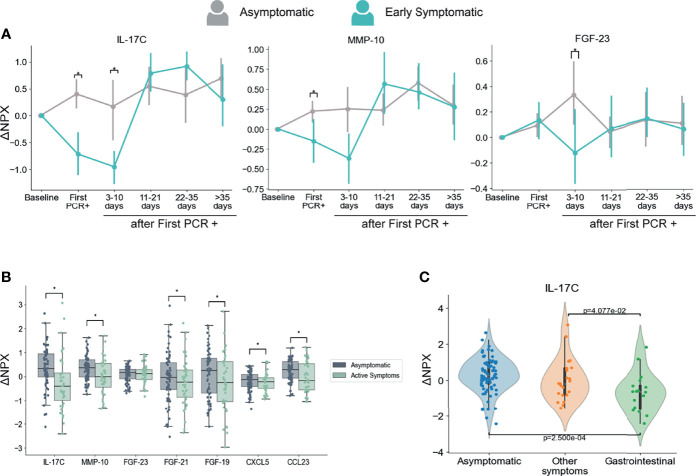
Dynamics of serum markers that are higher in Asymptomatic participants as compared with Symptomatic participants. **(A)** Mediators that are significantly higher in Asymptomatic participants in comparison with Early Symptomatic participants. Mean and 95% CI are indicated. **(B)** Mediators with decreased levels in Symptomatic participants at the time points when they had symptoms (Active Symptoms) than in Asymptomatic participants at any time point (Asymptomatic). This analysis includes only samples collected at PCR+ timepoints and compares levels of PEA markers regardless time after first PCR+. **(C)** IL-17C is differentially regulated in participants presenting with GI related symptoms (vomiting/nausea, diarrhea and/or abdominal pain) in comparison to participants that reported other symptoms, but none of them GI related, or to Asymptomatic participants. This analysis includes only samples collected at the time of first PCR +. *p < 0.05.

As anticipated, we found a strong association between the presence of symptoms and levels of multiple inflammatory markers. Interestingly, we also identified three immune mediators that showed significantly higher levels in the Asymptomatic group than in the Early Symptomatic group at the first PCR+ and/or at 3-10 days after infection. Those immune mediators are IL-17C, MMP-10, and FGF-23 (**Figure 5A**).

Next, we compared the PEA markers between all samples from symptomatic participants collected at the time they reported symptoms and were PCR+ (including Early and Late Symptomatic participants) and all samples from asymptomatic participants at the time they were PCR+. This analysis considers the presence or absence of symptoms regardless of the time after first PCR+. In agreement with our longitudinal analysis in Fig 5A, IL-17C and MMP-10 showed significantly higher levels in asymptomatic participants than in symptomatic participants at the time they reported active symptoms (**Figure 5B**). In addition, we also found significantly higher levels of CCL23, FGF-19, FGF-21 and CXCL5 in asymptomatic participants than symptomatic participants at the time point they reported symptoms (**Figure 5B**).

Moreover, SARS-CoV-2 infection induced early IL-17C, MMP-10, FGF-23 and CCL23 upregulation in Asymptomatic participants when compared to their baseline levels, while no significant changes, or a significant decrease in the case of IL-17C, was detected in Early Symptomatic participants (**Table S3** and **Figure S7**). These analytes therefore increased in serum early after infection in asymptomatic participants, yet unchanged or downregulated among Early Symptomatic participants.

IL-17C is a cytokine described as pro-inflammatory (50) and in combination with other mediators, works as an epithelial barrier against different bacterial (51, 52) and viral infections (53, 54). Interestingly IL-17C can be involved in tissue repair and protection of nerve fibers (54). CCL23, while also known as a pro-inflammatory chemoattractant (55) has been involved in angiogenesis by promoting migration of endothelial cells (56, 57). Other proteins in this group that could be related to tissue repair are the FGF-23, FGF-19 (also negatively correlated with number of symptoms, **Figure 3A**), and FGF-21 (58). CXCL5 is a neutrophil chemoattractant that has been shown to have important roles in homeostasis and wound healing (59, 60).

Given the importance of IL-17C in innate immunity and tissue repair, the clear contrast between the upregulation observed in Asymptomatic participants (**Figure S5**), the downregulation found in Early Symptomatic ones (**Figure 5**), and the negative correlation with the number of symptoms (**Figure 2A**), we sought to explore further other aspects of this cytokine. Specifically, we wondered what the mechanism of downregulation of this cytokine during symptomatic infections could be. In agreement with previous reports that indicate that IL-17C is not produced by hematopoietic cells (61), we did not find significant changes of the expression of IL-17C gene in the blood RNA-seq data from participants in the CHARM cohort in either the Asymptomatic or Early Symptomatic groups (**Table S4**).

The upregulation of IL-17C during respiratory viral infection might be produced by epithelial cells upon virus infection (62) which would explain the profile we observed in asymptomatic participants. Downregulation of IL-17C has been previously reported as a consequence of changes in the gut microbiota after treatment with antibiotics in a mouse model (63). Interestingly, COVID-19 patients have been previously reported to suffer microbiota composition modifications that are associated with the degree of severity (64, 65). Related to this, serum levels of IL-17C were found to be lower in participants with gastrointestinal (GI) symptoms than in those with no GI symptoms in hospitalized COVID-19 patients (66). To assess if there is an association between IL-17C and GI tract involvement in this cohort, we analyzed the levels of IL-17C among participants that reported GI symptoms (nausea/vomiting, diarrhea, and/or abdominal pain) at the time of first PCR+. Our results show that GI symptomatic group of participants presented significantly lower levels of IL-17C protein in the circulation in comparison to participants that presented only non-GI symptoms or those that were asymptomatic (**Figure 5C**), indicating a further association between GI symptoms and decreased levels of IL-17C. Therefore, it is possible that changes in microbiota during symptomatic infection modulates systemic circulating levels of IL-17C.

In conclusion, we identified a group of cytokines, including IL-17C, MMP-10, FGF-23, CCL23, FGF-19 and CXCL5 that are differentially regulated during asymptomatic and mild symptomatic infections and are known to be associated with tissue repair functions. We did not find significant changes in the expression of these genes in blood cells, suggesting that the expression of these proteins is regulated in tissues from respiratory or GI epithelia, and proteins are released to circulation. Given their differential patterns of expression regarding presence of symptoms and their previously described functions, we propose that they might have an important role in protecting the lung from tissue damage and subsequent clinical manifestation in asymptomatic individuals.

## Discussion

The immune response to severe COVID-19 has been well characterized, often by remarkable studies that included asymptomatic and/or mild symptomatic participants as control groups (11, 16, 19, 21, 67). To fully understand the pathogenesis of SARS-CoV-2 infection, it is also critically important to unravel the mechanisms that protect asymptomatic individuals from developing symptoms. Here, with a unique prospective study of healthy young adults with either mild or asymptomatic SARS-CoV-2 infection, we show the dynamics of the temporal immunity through the longitudinal analysis of the antibody response and a serum proteomic analysis.

The development of antibody responses and the levels of viral load as estimated by Ct value by PCR showed very similar profiles between asymptomatic and symptomatic participants. However, while the Ct dynamics were similar in participants with or without symptoms, slightly significantly higher viral load was found at the time of first PCR+ in symptomatic participants. Other studies have found an association between severity and viral load as well (68–71). The modest differences in our study as compared to other reports might be due to the presence of minor symptoms in many of the symptomatic participants in our cohort. We also found a positive correlation between the number of symptoms and viral load, which might explain the fact that several markers in the PEA analysis are correlated with both number of symptoms and viral load. It is well known that the innate immune response early during infection induces the B cell response and therefore is expected to contribute to the development of antibodies (72, 73). However, in this study we did not find any significant correlations between the levels of S IgG or neutralizing antibodies elicited and the relative serum levels of any of the PEA markers during the acute phase of the infection (data not shown) as reported before (12).

In patients with severe COVID-19, there is an abundance of cytokine production, that can induce a cytokine storm in addition to a series of adverse reactions (21, 38, 67). Here, we profiled the dynamics of the inflammatory response to SARS-CoV-2 in young adults with mild and asymptomatic infection, and identified three temporal waves of inflammatory markers. Overall, the participants of our cohort presented with upregulation of markers of severe disease reported before such as IL-6, IL-8 and CXCL10 (9, 10, 21), with most of them increasing early after infection and decreased over the time (see the first and second waves in **Figure 2**). In agreement with our results, the control of inflammatory response early in SARS-CoV-2 infection is crucial to avoid severe disease (9). However, it is important to mention that a subset of previously described severe disease markers, including IL-17A, CASP-8 and TNF-α, which belong to the third wave (**Figure 2A**), remained upregulated in our study until later time points post infection (≥ 11-35 days) (17, 67). These cytokines may act synergistically to promote an anti-viral response (74, 75) and may not be enough to define severe disease. Therefore, they should be used in combination with clinical and demographic information of the cohort.

We found multiple mediators positively correlated with number of symptoms that followed a temporal profile associated with symptom onset (**Figures 4**, **S4**), with highest levels at the peak of symptoms and a subsequent decline. As expected, several of these mediators were inflammatory markers such as IL-8, TNF-β, IL-18R1, and IL-22RA1. However, other mediators in this group can exert both inflammatory and immunoregulatory functions, such as sPD-L1 and IL-33 (**Figure 3A**). sPD-L1 can be induced by pro- and anti-inflammatory cytokines, but also directly by viral infections (76, 77). IL-33 on the other hand is an alarmin that was correlated positively with SARS-COV-2 and HIV specific antibody production (78, 79), and with anti-viral cytotoxic T cell response (80).

On the other hand, the cytokine IL-17C and FGF-19 were negatively correlated with symptoms, while CXCL5 and TWEAK were negatively correlated with viral load. In contrast to our findings, CXCL5, which was also downregulated after infection in our study (**Figures 2A** and **S3C**), was reported to be induced after *in vitro* SARS-CoV-2 infection of primary lung cells (62). TWEAK was also downregulated after infection in our study (**Figures 2A**, **3B**). However, it has been previously found elevated in SARS-CoV-2 patients (81), but decreased levels were found in patients infected with HIV (82).

**Figure 6:**
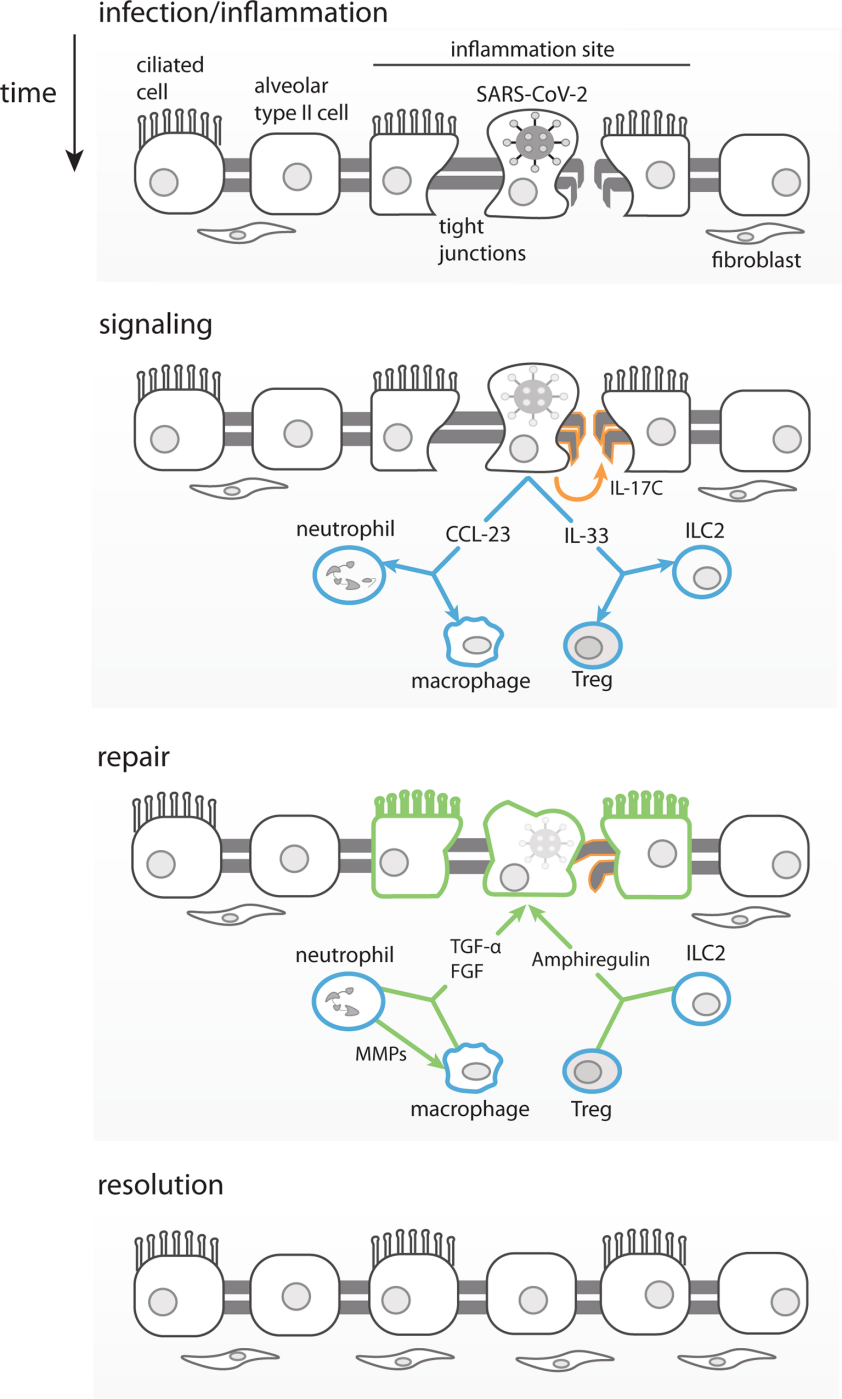
Proposed Model for Asymptomatic SARS-CoV-2 infection. Infected lung epithelial cells induce an inflammatory response and mild damage in the pulmonal tissue barrier through tight junction disruption. The immune mediators produced in this context, such as IL-33, can stimulate ILC2 and Tregs to produce amphiregulin and promote tissue repair. IL-17C produced by epithelial cells would also contribute to the lung repair, by stimulating tight-junction proteins production. Chemokines such as CCL23 once produced by infected epithelial cells would recruit neutrophils and promote local differentiation of macrophages. MMP-10 produced by neutrophils would alternatively activate macrophages. Both, macrophages and neutrophils produce FGFs and TGF-α leading to fibroblasts recruitment, growth and promoting lung epithelial barrier repair, respectively.

The fact that our correlation analysis revealed many analytes that are exclusively correlated with the number of symptoms, and not with viral load, may be attributed to an indirect induction of these markers, through an alternate pathway than the virus replication *per se*. sPD-L1 and IL-33 are probably induced by other mediators and not by viral replication, since they were not correlated with viral load. IL-33 was shown to be induced by IL-17A in γδ T cells in a mouse model of influenza infection (83). Both IL-17A and IL-33 were induced after infection here, but only IL-33 is positively correlated with number of symptoms. IL-33 is a growth factor that plays a major role in lung tissue repair by inducing the production of amphiregulin. IL-33 production is induced in influenza infected epithelial lung cells that in combination with IL-18, bind to their respective receptors (IL-18R and ST2) on regulatory T cells (Treg) (84) and/or innate lymphoid cells (ILC) (85) to promote lung tissue repair and inflammation control (**Figure 6**). Interestingly our results show that IL-33 is the analyte which is the most positively correlated with number of symptoms.

Our PEA analysis revealed, to the best of our knowledge for the first time, a group of analytes that shows lower serum levels in participants that reported symptoms at the time of diagnosis, as compared to those who remained asymptomatic during the study-namely IL-17C, MMP-10 and FGF-23. It was proposed that IL-17C would be able to boost IL-17A production to reinforce innate host barrier (61) during influenza infection for example (84, 85). In addition to being downregulated in early symptomatic participants (in both sexes when grouped separately), IL-17C was also negatively correlated with symptoms. Importantly, IL-17C, MMP-10, FGF-23 and CCL23 are upregulated only in asymptomatic participants early after infection, which strongly suggests their role in the control of COVID-19 clinical signs. The FGFs are involved in pulmonary tissue repair if signals of fibrosis occur (86) and in the negative modulation of inflammation (87, 88). MMP-10 was reported as a negative regulator of macrophage activation (89), indicating its role in the regulation of inflammatory response (90).

These results indicate that despite the mild disease in the CHARM cohort, individuals might be experiencing some degree of temporary tissue injury caused by the infection and/or by the inflammation induced after infection. It is possible that participants from our cohort presented lung injury related to COVID-19, but we cannot discard the possibility of gastrointestinal damage as well, since individuals also reported here and elsewhere (66) experiencing diarrhea, nausea and abdominal pain. An indicator that the symptomatic individuals might have lung and/or gut tissue damage related to COVID-19 is the high levels of TNFSF14 detected by PEA, and as reported before this protein is implicated in lung fibrosis when produced by local fibroblasts acting in tissue remodeling (91) but also play a role in limiting inflammation in an animal model of chronic colitis (92). It was shown by others that even asymptomatic SARS-CoV-2 infection can cause lung injury (19, 93), but the tissue protective factors may limit the extent of lung pathology and consequently the clinical symptoms in asymptomatic and mild symptomatic participants in this study. Moreover, many of the participants of this study reported shortness of breath, fatigue and/or cough, which could be symptoms of lung damage (94). It is possible that IL-17C, MMP-10, FGF-19, FGF-21, and FGF-23 act together to guarantee viral clearance and to promote lung tissue repair in asymptomatic individuals, and the early symptomatic individuals had a delay in this response. Moreover IL-33, TGF-α and IL-17A are all upregulated in our cohort, and may also play a role in tissue repair and control of inflammation (42, 74, 87, 95).

It has been reported that TLR activation can induce IL-17C (96), and it is mostly produced by epithelial cells in lung, skin or colon (96, 97), promoting tissue repair through an unknown mechanism that involves tight junction proteins (98, 99). Tight junction proteins, such as claudins and occludins are crucial for epithelial barrier function and are composed by several classes of cytosolic, transmembrane and cytoskeletal proteins, that regulate paracellular permeability, an important physiological condition to keep normal respiration (100, 101). Therefore, the pattern of expression of IL-17C that we found with regards to symptoms, and its previously described role in tissue repair, suggests that the modulation of this cytokine might have important implications in the control of symptoms in asymptomatic participants, and in the contribution to symptom resolution in early symptomatic participants. While the upregulation of IL-17C during respiratory viral infection might be produced by epithelial cells upon virus infection (62) the mechanisms for the downregulation of serum levels in Early Symptomatic participants are unclear. One possibility could be related to changes in the composition of their microbiota as a consequence of the SARS-CoV-2 infection. Decreased expression of IL-17C by GI epithelial cells has been found upon antibiotic-induced microbiota perturbations in animals (63). Importantly, the influence of SARS-CoV-2 infection in the gut microbiome composition has been reported by multiple groups (64, 65, 102). Moreover, associations of the microbiome composition with COVID-19 severity and inflammatory markers have been identified (64, 102), highlighting the importance of gut dysbiosis in the regulation of the immune response to respiratory viral infections thorough the lung-gut axis (103, 104). In our study, we found lower levels of IL-17C in participants with GI symptoms than in those with other symptoms, supporting an association between the decreased levels of IL-17C and GI involvement, possibly as a result of microbiota changes in the gut. In agreement with this hypothesis others have reported not only IL-17C decrease as a result of changes in the microbiota, but also CCL23, MMPs and FGFs (39, 63, 105, 106). Further research to clarify the mechanisms underlying the downregulation of IL-17C during symptomatic COVID-19 will have important implications in our understanding of COVID-19 pathogenesis, which is critical for clinical management and identification of new possible targets for treatment.

We propose that SARS-CoV-2 infected respiratory epithelial cells, in asymptomatic and otherwise healthy young adults, could produce IL-17C through TLRs activation. Importantly, expression of IL-17C has been shown to be induced in epithelial cells by SARS-CoV-2 (62, 97, 107), rhinovirus (108), and bacterial infection (97). The release of IL-17C could also be induced by IL-33 and IL-17A expressed by infected cells as part of the initial inflammatory response (see model in **Figure 6**). Infected epithelial cells would also release chemokines, such as CCL23 (109), that would recruit and promote the local differentiation of monocytes/macrophages and neutrophils, which would be the main producers of FGFs, MMP-10 and TGF-α. IL-17A, IL-17C and IL-33, among others, and would help promote viral clearance and control the inflammation. In parallel, the viral infection also causes tissue damage (of lung, guts, etc.) that is repaired by IL-17C through induction of tight junction proteins, in combination with FGFs and TGF-α that would exert fibroblasts recruitment and proliferation to ultimately resulting in tissue repair (**Figure 6**).

A cross-sectional study which also focused on asymptomatic SARS-CoV-2 infections, found preferential expression of growth factors and an immune tolerance profile in blood from asymptomatic as compared to symptomatic individuals (16). Importantly, this study also found a stronger virus-specific Th17 response in asymptomatic as compared to symptomatic participants, while Th1 and Th2 responses were similar in the two groups. It is possible that the higher levels of IL-17C in asymptomatic infections could promote the establishment of robust virus-specific Th17 responses.

Our study has an important advantage due to the use of baseline samples obtained from participants prior to infection, allowing us to study differences in immune response that occur during infection while minimizing other confounders that could induce immune mediators in the participants before infection. In fact it is known that intense physical training, which is an important component of the basic training of Marine recruits, can contribute to changes in inflammatory markers (110). To account for these possible changes due to training effects, we incorporated an uninfected control group with 3 longitudinal samples in a period of 56 days. Indeed, the control group presented significant enhancement in IL-6 at 56 days after enrollment in this study, as well as variations in other cytokines such as MCP-3, NT-3, and CASP-8 (**Table S1**) all in different time points. Another advantage of our study is that the frequent sampling and follow up allowed for identification of a high number of asymptomatic cases, which are usually difficult to detect. We are confident that the asymptomatic participants did not develop symptoms during the course of their infection since any symptoms that might have started between study visits were specifically asked about on the questionnaire. In addition, sample and data collection was conducted by the medical research team in the same location where the participants reside for their basic training at MCRDPI. If symptoms developed, the study team was made aware by medical providers. Therefore, it is highly likely that if a participant experienced any symptoms during the course of the illness, they were identified and the complaints recorded by the study team.

This study has some limitations. For example, we did not have access to participants’ PBMCs or lung epithelial cells to perform a deeper analysis to understand better the mechanisms underlying the mild and asymptomatic COVID-19. Given the characteristics of the cohort (mainly white, young adults), these results might not be representative of children or older adults, of other races/ethnicities, or cases with severe COVID-19. Additionally, the female representation in the cohort was low (11.7%) and the sample size is still modest to being able to analyze the data stratifying it per symptom. Despite these challenges, we were able to find an important set of markers associated strongly with asymptomatic infection or protection from symptomatic disease. Another potential limitation is the possible presence of other respiratory infections during course of the study. However, long-term passive surveillance data, collected from January 1, 2017 to December 31, 2020, indicated a low level of the circulation of non-SARS-CoV-2 respiratory infections at MCRDPI during the time the CHARM study took place, which was dramatically reduced in 2020 as compared to previous years (unpublished data). The decrease in overall respiratory infections was probably due to the safety measures and protocols established to reduce SARS-CoV-2 spread. In addition, the timeline of the CHARM study did not overlap with the influenza season, and therefore the probability of influenza virus infections interfering with our results is minimal (111, 112). Our data depended on serial samples obtained before and during documented SARS-CoV2 infections that occurred in nearly half the participants during an 8-week study period (22). Given the high rate of SARS-CoV-2, the serial sampling and low rate of other respiratory illnesses during the study period we believe that our findings were due to SARS-CoV-2 and not the effects of other circulating viruses.

Collectively, our results show a group of immune mediators that may be pursued as potential targets for developing therapies as well as prognostic testing. To our knowledge this is the first study that shows the immune longitudinal profile of asymptomatic individuals, spanning their baseline state prior to infection through viral clearance, in combination with potential markers that are inhibited in mild symptomatic COVID-19.

## Data Availability Statement

The RNA-seq data presented in the study are deposited in the Gene Expression Omnibus repository, accession number GSE198449. The PEA data are available in **Table S5**.

## Ethics Statement

The studies involving human participants were reviewed and approved by NMRC.2020.0006. The patients/participants provided their written informed consent to participate in this study.

## Author Contributions

AS-S: performed serological assays, coordinated PEA data generation, analyzed data, prepared figures, wrote the manuscript; NS: performed statistical and data analysis, prepared figures, and wrote the manuscript; CWG, DLW, SL, RL: contributed to sample and data collection; SP and NK: performed neutralization assays; SV, SM, NM and CMM: contributed to sample preparation for PEA OLINK and serological assays; YG: contributed to data management and data analysis; VDN: supervised RNA-seq assays; XBY: contributed to sample management; M-CG: contributed to projects management; AC: prepared figures; WC, EZ: Contributed to data analysis; AB: supervised data generated from neutralization assays; OGT: supervised data analysis and contributed to scientific discussions; AGL: supervised sample and data collection and contributed to scientific discussions; SCS: supervised data generation, and data analysis and manuscript preparation; IR: analyzed data, prepared figures, wrote the manuscript, and supervised data generation, data analysis and manuscript preparation. All the authors reviewed and edited the manuscript.

## Funding

This work received funding from the Defense Health Agency through the Naval Medical Research Center (9700130), from the Defense Advanced Research Projects Agency (contract number N6600119C4022), from the National Institutes of Health (R01GM071966), and from the Simons Foundation (395506).

## Author Disclaimer

CWG, DLW, SL, RL and AGL are military Service members or U.S. Government employees. This work was prepared as part of their official duties. Title 17, U.S.C., §105 provides that copyright protection under this title is not available for any work of the U.S. Government. 355 Title 17, U.S.C., §101 defines a U.S. Government work as a work prepared by a military Service member or employee of the U.S. Government as part of that person’s official duties. The views expressed in the article are those of the authors and do not necessarily express the official policy and position of the US Navy, the Department of Defense, Uniformed Services University, the US government or the institutions affiliated with the authors.

## Conflict of Interest

The authors declare that the research was conducted in the absence of any commercial or financial relationships that could be construed as a potential conflict of interest.

## Publisher’s Note

All claims expressed in this article are solely those of the authors and do not necessarily represent those of their affiliated organizations, or those of the publisher, the editors and the reviewers. Any product that may be evaluated in this article, or claim that may be made by its manufacturer, is not guaranteed or endorsed by the publisher.
