# Supplementary material for: Asymptomatic SARS-CoV-2 Infection Is Associated With Higher Levels of Serum IL-17C, Matrix Metalloproteinase 10 and Fibroblast Growth Factors Than Mild Symptomatic COVID-19
Source: Front Immunol. 2022 Apr 5;13:821730. doi: 10.3389/fimmu.2022.821730 (PMC9037090; doi:10.3389/fimmu.2022.821730)

Supplementary Material

1. Supplementary Tables and Figures

Table S1. Longitudinal PEA results of all analytes measured for all infected participants. Each time point was compared to the corresponding group of baseline values using a paired t-test, and p-values were then adjusted using the Benjamini-Hochberg procedure. The fold change, computed as the difference of the means of the two sets, is also reported.

Table S2. Longitudinal PEA results of all analytes measured comparing Early or Late Symptomatic groups with Asymptomatic in all time points. P-values were computed using a Mann-Whitney test and multiple hypothesis correction was performed with the Benjamini-Hochberg procedure. Fold change was computed as the difference of the means of the symptomatic group and the asymptomatic group, so a negative value implies higher analyte levels in asymptomatic participants.

Table S3. Longitudinal PEA results for some analytes of interest. Comparison was made between each group of participants (Early and Late Symptomatic & Asymptomatic) with their baseline results.

Table S4. Longitudinal differential expression analysis for select genes in whole blood samples for Asymptomatic and Early Symptomatic groups of participants. NaN: adjusted p-values can be set to NaN for three reasons: 1. all sample counts are zero, 2. there is an extreme outlier in sample counts, or 3. mean sample count is below the threshold for automatic filtering.

Table S5. NPX values from the Protein Extension Assay.

Table S6. Metadata associated with samples included in the PEA assays. Longitudinal data for SARS-CoV-2 PCR (Ct values for N, S, and ORF1ab genes) and symptoms for participants with PEA data is provided.

* Please note that all supplementary tables are in tabs in an excel file.

Figure S1. Symptomatic and Asymptomatic SARS-CoV-2 viral load and sex differences in viral load and serology. **(A)** Longitudinal ORF1ab and N genes PCR test results, with loess curve and 95% confidence interval (gray shedding) **(B)** Ct values in the groups of symptomatic (n=85) and asymptomatic participants (n=64). **(C)** Sex differences for viral load at first PCR+ (S, ORF1ab and N genes) (n=4 asymptomatic females, n=12 symptomatic females, n=81 asymptomatic males, n=52 symptomatic males) **(D)** S-specific IgG titers (n=84 asymptomatic, n=55 symptomatic) and half inhibitory infectious dose (ID50, n=44 asymptomatic and n=34 symptomatic participants) at 10-63 days after infection. **(E)** S IgG titers in females (F, n= 14) and males (M, n=119) and ID50 in females (n=12) and males (n= 31) at 10-63 days after infection. Mean ± standard deviation is indicated in C, D and E.

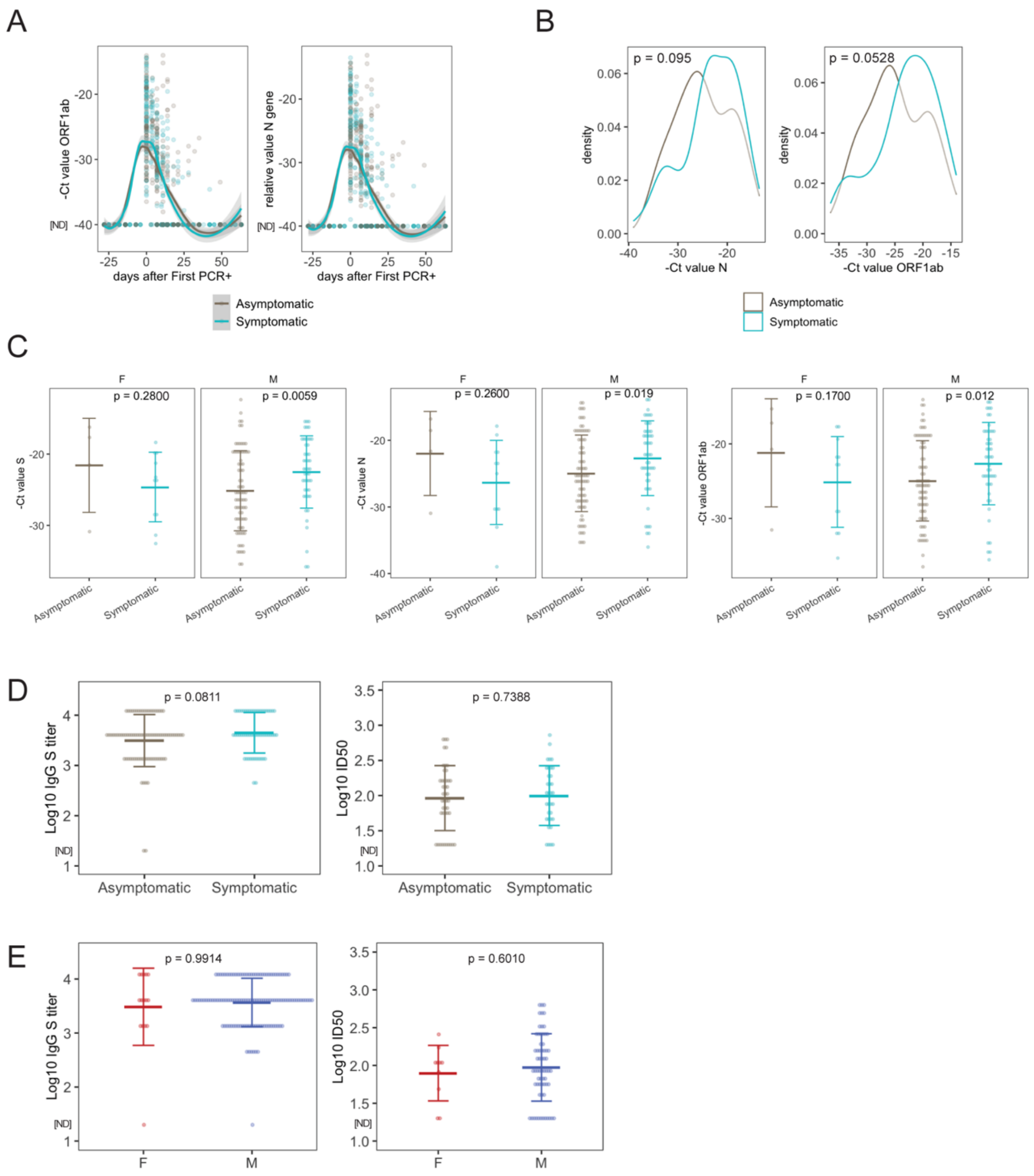

Figure S2. Estimated cell proportions from blood bulk-RNAseq data from SARS-CoV-2 infected participants overtime (n=240 participants). Mann-Whitney U test was used for comparisons between the "Before" infection samples and each later time point. *p<0.05, ** p<0.01, *** p<0.001.

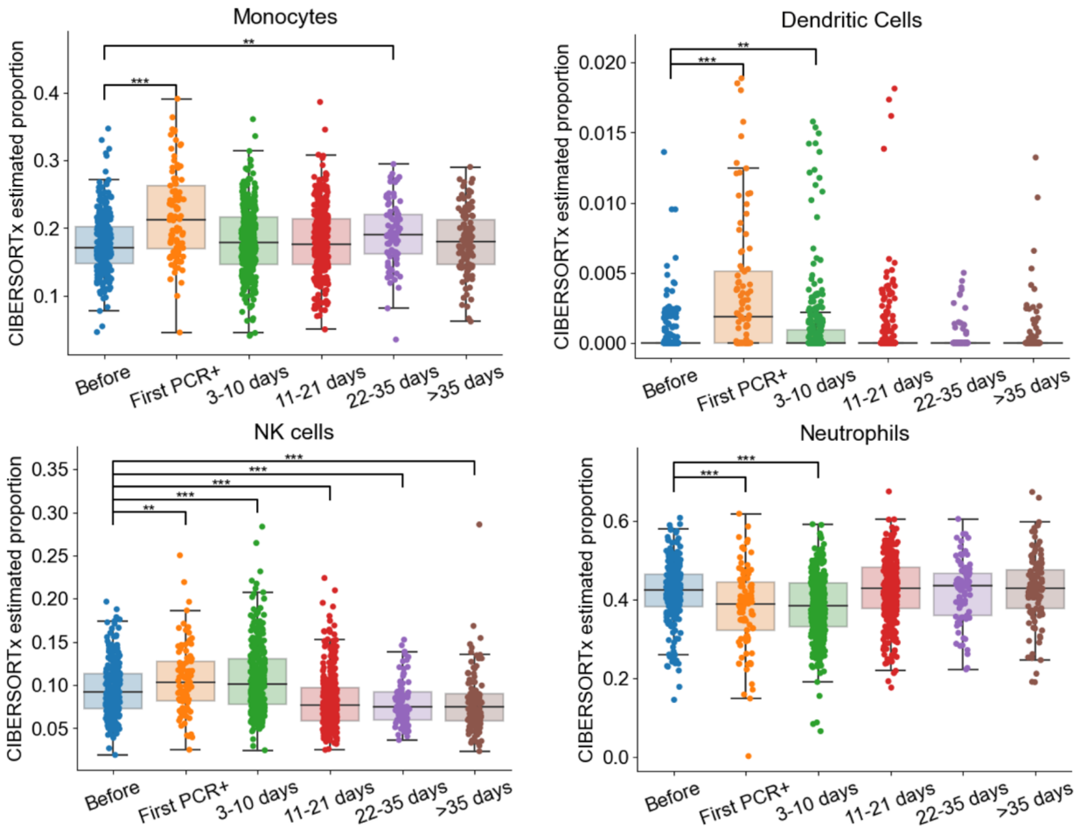

Figure S3. Longitudinal levels of mediators in Late Symptomatic participants in comparison to Asymptomatic participants. **(A)** Distribution of number of symptoms over time in Late Symptomatic group of participants. **(B & C)** Markers that are shown in Figures 4 & 5 for Early Symptomatic are shown here for Late Symptomatic group. Mean and 95% CI are indicated.

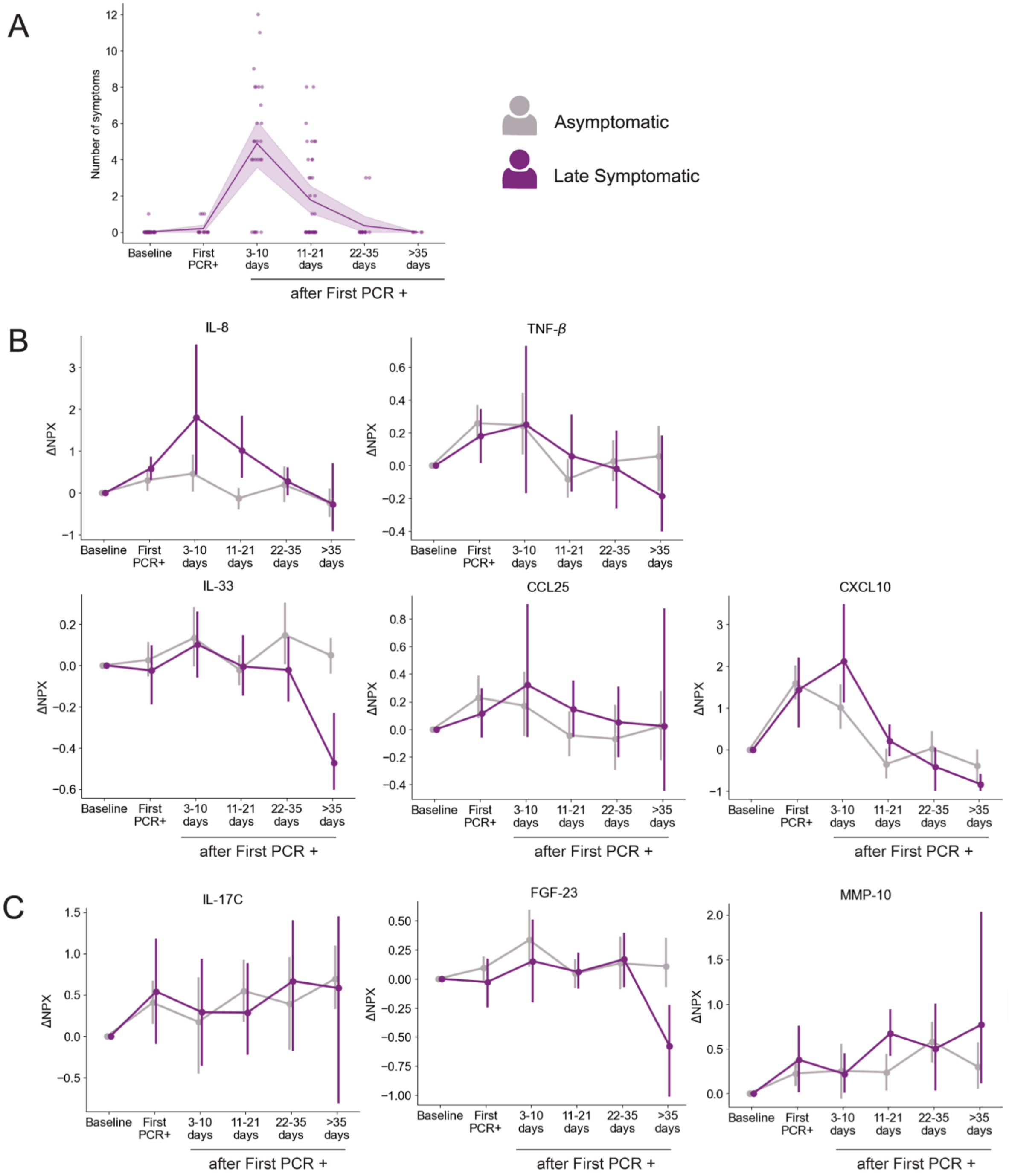

Figure S4. Levels of serum mediators show a temporal association with symptoms. **(A)** Several inflammatory markers show peak levels at the time of maximum number of symptoms. **(B)** Increased levels of selected mediators in symptomatic participants in samples collected at time they report symptoms (Active Symptoms) than in asymptomatic participants at any time point (Asymptomatic). This analysis includes only samples collected at PCR+ timepoints and compares levels of PEA markers regardless time after first PCR+. *p<0.05.

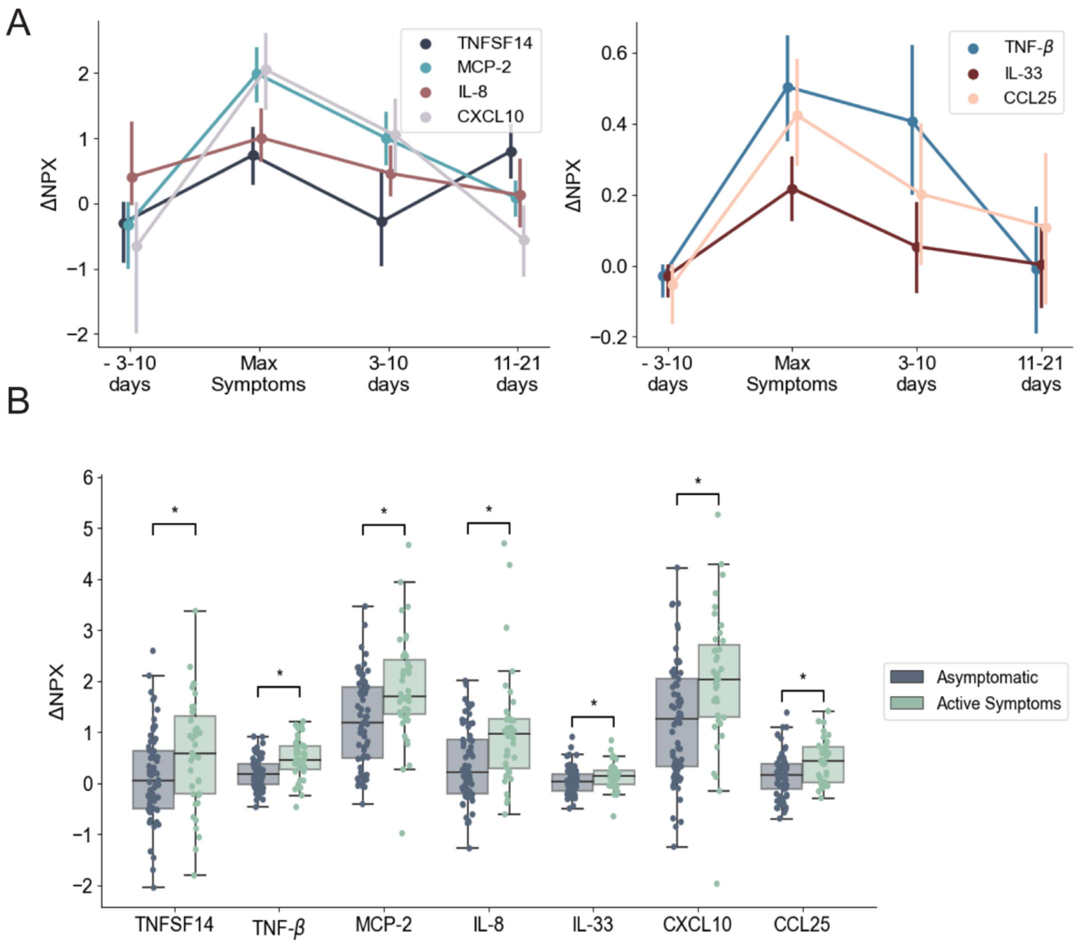

Figure S5. PEA results by Sex differences and symptoms. Males and females were grouped as Symptomatic and Asymptomatic (asymptomatic females n=2, males n=44; symptomatic females n=10, males n=33). *p<0.05.

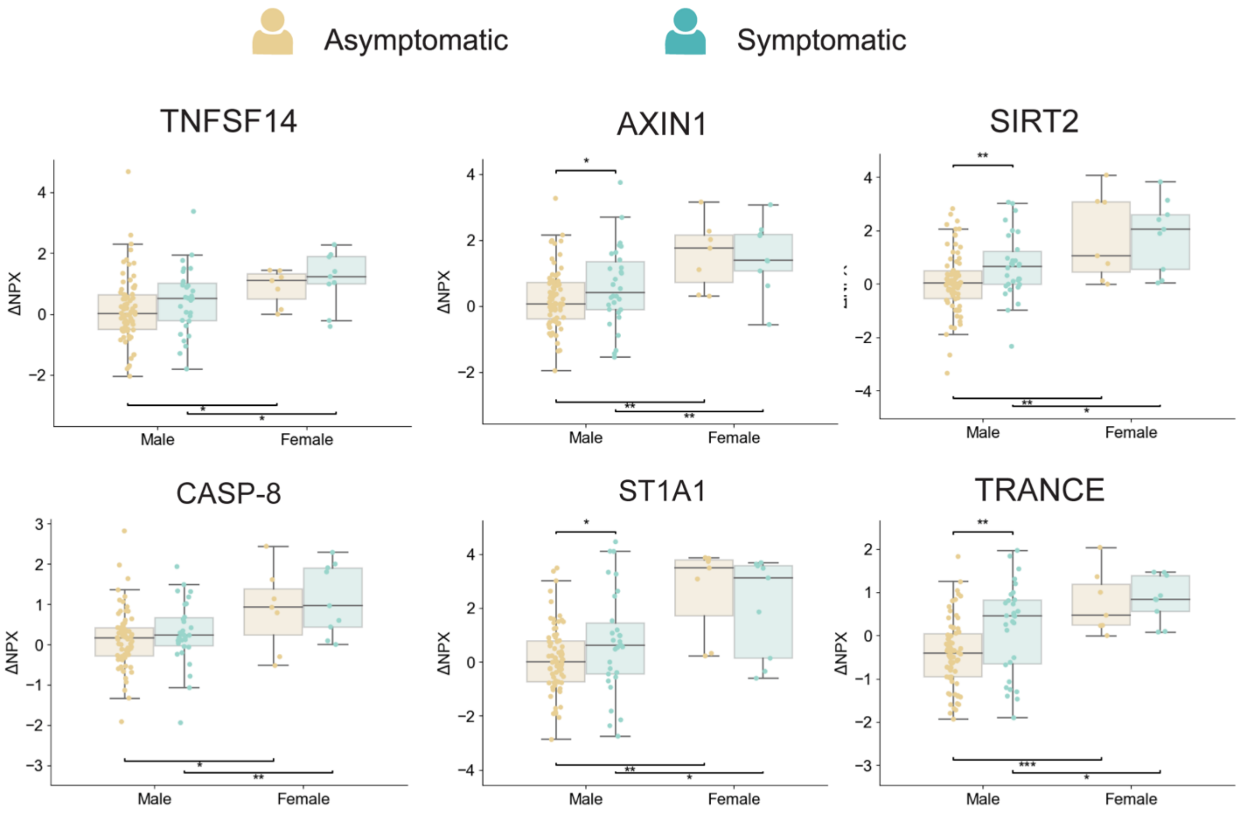

Figure S6. Estimated cell proportions from blood bulk-RNAseq data from Asymptomatic (110 participants) and Early Symptomatic SARS-CoV-2 (77 participants) infected participants overtime. Mann-Whitney U test was used for comparisons between the two groups at each time point. *p<0.05, ** p<0.01, *** p<0.001.

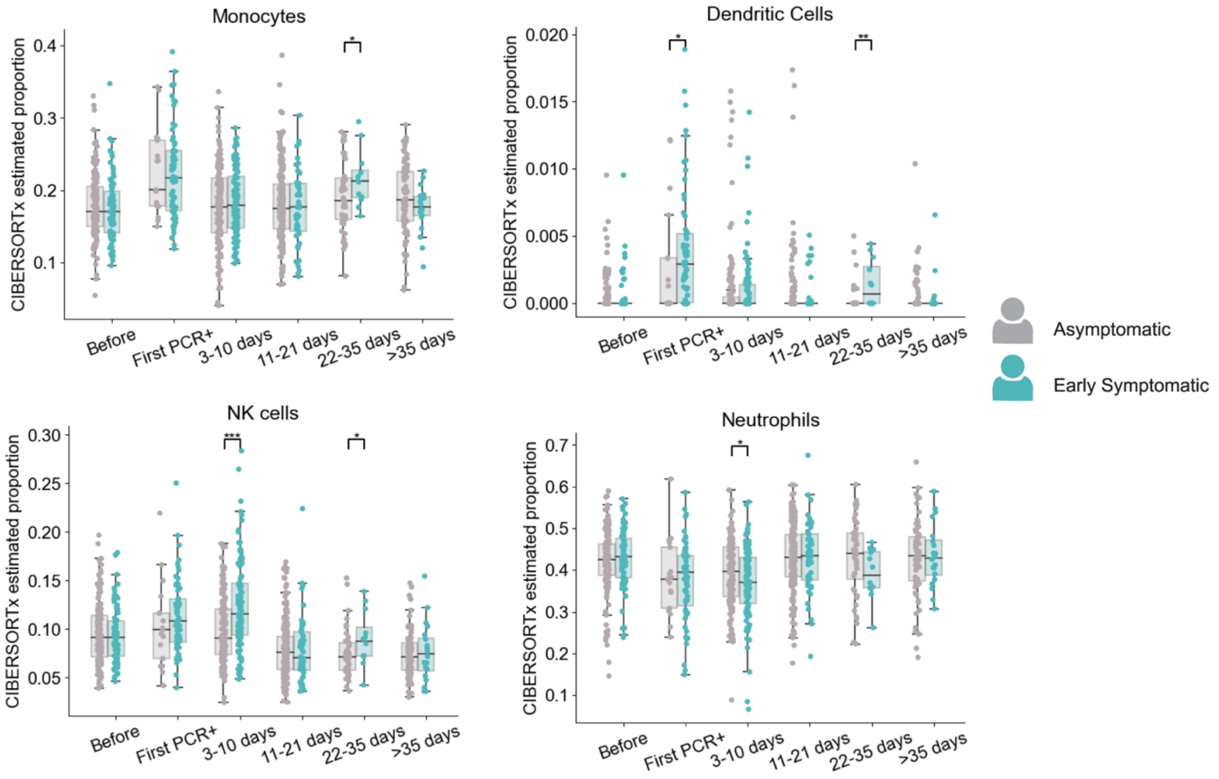

Figure S7. Levels of IL-17C, MMP-10, FGF-23 and CCL23 are increased in serum in Asymptomatic participants at the time of First PCR+ or 3-10 days after First PCR+, but not in Early Symptomatic participants. *p<0.05.

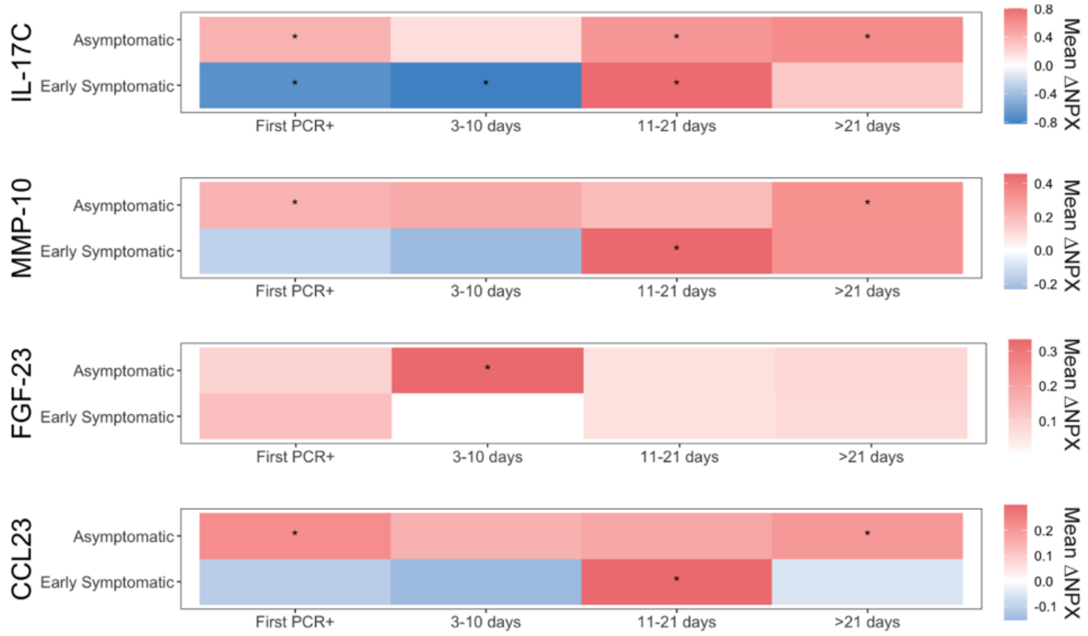

Supplement: Supplementary file 1 [file DataSheet_1.pdf]
